# Baseline and post-stress seasonal changes in immunocompetence and redox state maintenance in the fishing bat *Myotis vivesi*

**DOI:** 10.1371/journal.pone.0190047

**Published:** 2018-01-02

**Authors:** Ulalume Hernández-Arciga, L. Gerardo Herrera M., Alejandra Ibáñez-Contreras, Roxana U. Miranda-Labra, José Juan Flores-Martínez, Mina Königsberg

**Affiliations:** 1 Posgrado en Ciencias Biológicas, Instituto de Biología, Universidad Nacional Autónoma de México, Ciudad de México, México; 2 Laboratorio de Bioenergética y Envejecimiento Celular, Departamento de Ciencias de la Salud, Unidad Iztapalapa, Universidad Autónoma Metropolitana, Ciudad de México, México; 3 Estación de Biología Chamela, Instituto de Biología, Universidad Nacional Autónoma de México, San Patricio, Jalisco, México; 4 Laboratorio de Neurofisiología, Applied Research in Experimental Biomedicine S.A. de C.V. (APREXBIO), Ciudad de México, México; 5 Unidad de Experimentación Animal, Biología Integral para Vertebrados (BIOINVERT®), Estado de México, México; 6 Laboratorio de Fisiología Celular, Departamento de Ciencias de la Salud, Unidad Iztapalapa, Universidad Autónoma Metropolitana, Ciudad de México, México; 7 Laboratorio de Sistemas de Información Geográfica, Departamento de Zoología, Instituto de Biología, Universidad Nacional Autónoma de México, Ciudad de México, México; Institute of Zoology, CHINA

## Abstract

Little is known of how the stress response varies when animals confront seasonal life-history processes. Antioxidant defenses and damage caused by oxidative stress and their link with immunocompetence are powerful biomarkers to assess animal´s physiological stress response. The aim of this study was A) to determine redox state and variation in basal (pre-acute stress) immune function during summer, autumn and winter (spring was not assessed due to restrictions in collecting permit) in the fish-eating Myotis (*Myotis vivesi*; Chiroptera), and B) to determine the effect of acute stress on immunocompetence and redox state during each season. Acute stress was stimulated by restricting animal movement for 6 and 12 h. The magnitude of the cellular immune response was higher during winter whilst that of the humoral response was at its highest during summer. Humoral response increased after 6 h of movement restriction stress and returned to baseline levels after 12 h. Basal redox state was maintained throughout the year, with no significant changes in protein damage, and antioxidant activity was modulated mainly in relation to variation to environment cues, increasing during high temperatures and decreasing during windy nights. Antioxidant activity increased after the 6 h of stressful stimuli especially during summer and autumn, and to a lesser extent in early winter, but redox state did not vary. However, protein damage increased after 12 h of stress during summer. Prolonged stress when the bat is engaged in activities of high energy demand overcame its capacity to maintain homeostasis resulting in oxidative damage.

## Introduction

Animals confront stressful situations throughout their lives, whether due to changes in environmental conditions or to predation and competition [1,2]]. Environmental stress has a significant impact on evolutionary and ecological processes that affect and shape population genetic structure and evolution [3]. The former situations are potentially amplified by human activities due to habitat loss or alteration [4], causing rapid and often stressful and deleterious changes. Therefore, assessing animal responses to stress has a practical relevance for determining the impact of human activities (e.g. ecotourism, recreational fishing, vehicle traffic), or conservation interventions (e.g. animal trapping, restraint and handling, trans-location and radio-collaring, and species management programs) on animal well-being [5,6].

Different biomarkers to assess animal´s health in the wild have been reported; however, hormones dynamics, metabolic rate, and the magnitude and intensity of the immune response have become the gold standard of field ecologists [7–9]. Recently, determining redox state changes has also become an important tool in ecological studies [10]. Animals use oxygen to produce energy, which ultimately leads to production of free radicals along with non-radical reactive oxygen species, collectively called reactive oxygen species (ROS). These molecules induce cellular damage, such as lipid peroxidation, protein oxidation, and nucleic acid damage [11–16]. Enzymatic antioxidants, such as Superoxide Dismutase (SOD), Catalase (CAT), Glutathione peroxidase (GPx) and, along with the three-peptide Glutathione (GSH), are the main line of defense promoting ROS removal [17]. Nevertheless, certain cellular functions, such as redox signaling [18], rely upon ROS; hence, antioxidants maintain an optimum oxidant level rather than eliminate them entirely [19].

The importance of the immune response as a powerful biomarker to asses stress relies on the fact that changes in the endocrine system after stress episodes have paramount consequences on animal immune function. Stressful events, such as environmental condition fluctuations [20–23], cold temperatures [24–29] and lack of food [30–32] also affect an animals immunity, making this marker very useful to assess stress [33]. Furthermore, modifications in redox state have an important role during an immune response. Animal metabolic rate is usually elevated during the immune response [34], due in part to higher mitochondrial activity and consequently correlated with increased ROS production [35]. Additionally, leukocytes involved in the immune response, in particular neutrophils, produce ROS to eliminate pathogens [36] and to enhance T-lymphocytes activation [36,37]. Thus, assessing both redox state and immunocompetence provides a more integrated outlook of animal health state. Although several studies have measured antioxidant response [38–44] and immune variation [45–49] in wild animals during different seasons, they have used baselines measurements, and research on the effect of acute stress in these physiological parameters throughout the year is mostly inexistent. There are some experiments where redox changes [50] and cellular immunity [51–53] after acute stress have been determined under laboratory experimental conditions and some others where the effect on humoral immune response has been reported in the wild [9,54,55]. However, the results of these studies are contradictory. These contradictions probably arise from the differences in sample collecting conditions and the season when the samples were collected. Animals are engaged in different activities through the year with contrasting energy demands (i.e reproduction in spring/summer, or torpor during winter, etc.) and are exposed to different environment conditions (i.e high or low temperature, storms, lack of food, etc.) that might affect their redox state and immunocompetence. Accordingly, in this study we tested if the response to acute stress is related to the environment conditions when the stressful stimuli is presented and consequently to the season when it was measured, taking into account individual´s body condition, sex and reproductive state. We conducted this study with the Myotis fishing bat *Myotis vivesi*, Menegaux, 1901, endemic to the Gulf of California, Mexico. Ambient temperature (T_a_) in the islands where this bat roosts range from 45ºC in summer to 5ºC during winter [56]. During daytime, bats roost under rocks, which barely provide them minimal isolation from T_a_ [57]. Lactation in this bat occurs during summer, and during winter these bats use torpor [57]. Torpor increases the risk of oxidative stress damage during the rewarming periods, so that the animals adapted to this process must have strong antioxidant mechanisms [58]. Thus, *M*. *vivesi* represents an ideal natural model to test the following objectives: A) to determine seasonal variations in redox state and basal (pre-stress stimulus) immune function, and B) to determine the effect of acute stress on immunocompetence and redox state in different seasons. Redox state was determined by measuring systemic antioxidant enzymatic activity (AEA) and cellular damage (protein carbonyls). Bat immunocompetence was determined by measuring the cellular immune response (Phytohaemagglutinin test- PHA) and humoral immune response (bactericidal activity of plasma- BA). Bats were subjected to 6 and 12 h of movement restriction as a source of stress, and the change in their physiological parameters was monitored after each stress period.

Our results showed that the basal immune response was an important physiological response that changed through the year, with a trade off in functionality of immune components. The cellular immune response was higher during winter whilst the humoral response was higher in summer. Humoral response increased after 6 h of movement restriction stress and returned to baseline levels after 12 h. Basal redox state was maintained through-out the year, with no significant changes in protein damage, and the antioxidant activity increased during high temperatures and decreased during windy nights. Bats responded to the 6 h acute stress by enhancing their antioxidant activity especially during summer and autumn, and to a lesser extent in early winter, with no changes in redox state. However, some protein damage began to accumulate after 12 h of movement restriction stress during summer, when more than one energy demanding process concur, (i.e. humoral immune response, AEA response, thermoregulatory demands due to high temperatures, and lactation). All the above suggests that stress overload during highly energetically-demanding periods might force the system and produce cellular damage.

## Materials and methods

### Chemicals

All chemicals and reagents were purchased from Sigma Chemical Co. (St. Louis, MO). The reagents obtained from other sources are detailed throughout the text.

### Study site

This study was conducted in Partida Norte Island (28º52’30”N, 113º21’7”W), a 1.4-km^2^ island located in the midriff region of the Gulf of California, Mexico [59]. This island is home to the largest known colony of *Myotis vivesi* (~8,000 adults)[60]. Fieldwork was carried out for five days during each visit for two consecutive periods. The first period was during July, October and December 2014, and February 2015. The second period was during June and October 2015, and February 2016. Those months were selected in order to sample animals in summer when lactation occurs, in winter when bats use torpor, and in autumn, a transition period between these two activities. We did not sample in spring when the colony is mostly composed of pregnant individuals [61] due to restrictions in our research permit.

### Sample collection

Only adult bats were captured between 6:00–7:00 am directly from their roost site under rockslides. They were kept individually in small cotton bags, where the acute stress stimuli consisted of their immobilization within the bag. It has been reported that capture, handling and immobilization induces stress as shown by increase in glucocorticoid levels [62–65]. We also measured cortisol levels in fecal samples collected ≤ 6 h and ≥ 7 h after the animal was captured. Based on observations of gut transit time in our focal species, we considered that feces collected in the first period were formed before capture; thus, the effect of capture stress on cortisol levels is most likely reflected on feces collected in the second period. Accordingly, feces collected in the first period had significantly lower cortisol levels than those collected in the second period (data not shown). A basal blood sample (150 μl) was obtained from all bats after capture by bleeding from the right forearm vein. Afterwards, bats were randomly selected to form two groups: animals stressed for 6 h and animals stressed for 12 h. The second blood sample (150 μl) was drawn from the left forearm vein 6 or 12 h after capture, depending on the animal group. Blood was drawn by punctuation with a hypodermic needle (27Gx 13mm BD), collected in heparinized capillary tubes and placed in a 1.5 mL eppendorf tube. After 24 h, bats were returned to the place where they were captured. All samples were kept on ice during collection and handling, and plasma and erythrocytes were separated after centrifugation at 6000 rpm (Digital ZipSpin Centrifuge LWScientific), stored in 0.2 mL tubes and frozen in liquid nitrogen. Afterwards, samples were transferred from liquid nitrogen to dry ice and shipped to the laboratory where they were stored at -80ºC until their analysis.

We measured body mass with a portable XSXScale ES200G x 0.01G (± 0.01g) and forearm length with Vernier calipers (Mitutuyo CD-6´´CSX; ± 0.01mm). Sex was recorded for all bats captured. Only adult animals were used and they were differentiated from young individuals by examining their ossification index. Accordingly, we exposed the wing of each individual to transillumination using a headlight; in young individuals, the cartilaginous zone of the long phalanges is visible because less mineralized tissue allows more light to pass through and thus appears lighter than bone. When bats reach adulthood, epiphyseal plates eventually close and are no longer visible to the unaided eye. Fur color was also an indicator of age; adults have brown-gold fur while young have gray fur [66]. A total of 116 bats were captured during the first period of sampling (P1) (summer: 32 females, 6 males; autumn: 11 females, 5 males; early winter: 24 females, 11 males; late winter: 13 females, 14 males) and 117 during the second period of sampling (P2) (summer: 34 females, 6 males; autumn: 24 females, 13 males; late winter: 26 females, 14 males).

This study was carried out in strict accordance with the recommendations and permits approved by Mexican Government (Secretaría de Gobernación #013/13) and from Dirección General de Vida Silvestre (#01947/13), Mexico. All sampling procedures and experimental manipulations were performed according to the Principles of the Mexican Official Ethics Standard 062-ZOO-1999 and were approved as part of obtaining this permit. No other approval was required to conduct the study, as there is no IACUC/animal ethics board at our institution.

### Weather data

Data for ambient temperature (T_a_) and wind speed (W) were obtained from the nearest meteorological station in Bahía de los Ángeles, Baja California, Mexico. Data was registered every 10 sec. Considering that the rock roost does not completely insulate bats from T_a_ [57], we used the total daily data recorded to obtain the daily median ambient temperature (dŦ_a_) of each sample day. Considering that bats are mostly affected by wind during their foraging hours, we used the data recorded between 19:00 and 23:59 hours to obtain the median night wind speed (Ŵ_n_) of the sampled days.

### Body condition determination

Ecologists have measured body condition as a non-destructive method to estimate nutritional state and provide a snapshot of an animal's physiological state [67]. Here, body condition was determined with two methods. First, we used the Scaled Mass Index (SMI) [68] which relies on measures of body mass and linear measures of body size to calculate a condition index with the following formula:

^Mi=Mi[L0/Li]bSMA

where Mi is the weight (g), L_i_ is the forearm length (mm), L_0_ is the forearm arithmetic mean, bSMA is the scaled exponent estimated using online software [69], and ^Mi is the predicted body mass for an individual when the lineal body measurement is standardized to L_0_. The second method used to calculate body condition was the hematocrit percentage (%H). Hematocrit is defined as the percentage of the total blood volume occupied by erythrocytes, which depends on the variation in plasma volume, the rate of erythrocyte production and destruction, dehydration, toxins, and direct blood loss, and it may hence be used as an index of the ‘health’ of the oxygen transport system. It was calculated by dividing the total blood draw volume by the erythrocyte volume of each blood sample. This was done with the basal blood samples and the post stress blood samples (6 and 12 h).

### Phytohemagglutinin challenge

The delayed cutaneous hypersensitivity response was quantified as an indicator of cellular immunity responsiveness [70–72]. This response was assessed by injecting 50 μL of a phytohemagglutinin (PHA) solution (3 mg PHA/mL of phosphate buffered saline-PBS) on the right foot, and 50 μL of PBS on the left foot. PHA influences a variety of cell types and, therefore the response to PHA injection is complex, but can serve as an index for heightened immune cell activity [73,74]. Thickness of the foot was measured before injection and 6, 12 and 24 h afterwards using digital calipers (Mitutuyo CD-6´´CSX (± 0.01mm). Cellular immune response was calculated as the change in thickness of the PHA injected foot minus the change in the control foot [75,76]. Larger localized swelling indicates increased immune activity. Measurements were made in triplicate and the mean was used for analyses.

### Bactericidal activity (BA)

The antimicrobial capacity of plasma was assessed with the Liebl and Martin Ii protocol [77] as a measurement of serological components [78] (non-specific antibodies [79], complement cascade [80,81], and lysozyme activity [82]). Before assay, the bacteria *Escherichia coli* (ATCC #8739) were reconstituted according to manufacturer instructions. Stock solution of the bacteria was diluted to 1 x 10^5^ microbes mL^-1^. Plasma was diluted in sterile PBS 1:23, 25 μL of working solutions was added, and samples were incubated for 30 min at 37ºC. After the first incubation, 500 μL of Soy Broth (TSB) was added. Samples were incubated a second time at 37ºC for 12 h. After the second incubation the samples were analyzed spectrophotometrically (Beckman DU-650) at 340 nm. The portion of killed bacteria was calculated as 1- (Sample Abs_340_ / control Abs_340_). All samples were analyzed in duplicate.

### Protein extraction

Erythrocytes samples were first washed twice with 0.9% NaCl by centrifugation at 3000 rpm for 10 min. Protein was extracted afterward by adding lysis buffer (100 μL DTT 1M, 100 μL Phenylmethylsulfonyl flouride (PMSF) 0.1M, 1 cOmplete^TM^ tablet, 10 mL T-PER) and centrifugated at 13500 rpm at 4ºC for 15 min. Protein samples were separated in four aliquots to prevent frizzing and thawing. Before each assay, total protein concentration in each aliquot was determined spectrophotometrically at 595 nm using a commercial Bradford reagent (Bio-Rad, Hercules, CA, USA) [83].

### Antioxidant enzyme activity

Antioxidant enzyme activity (AEA) was analyzed spectrophotometrically (Thermo Scientific™ GENESYS 10S UV-Vis; Madison, WI USA) in erythrocyte protein samples as described elsewhere [84]. Briefly, SOD activity was determined through the xantine/xantine oxidase system, based on protocols by Paoletti *et al* [85]. The superoxide anion formed through this system reacts with the nitro blue tetrazolium (NBT) and generates a formazan salt that was measured spectrophotometrically at 560 nm every 30 sec for 5 min. One unit of enzymatic activity in this assay is considered as the amount of SOD needed to inhibit 50% of the superoxide reaction with NBT. CAT activity was quantified using the protocol established by Abei [86], which evaluates the decline in absorbance at 240 nm, as H_2_O_2_ is catalyzed to H_2_O and O_2_, every 15 sec for 3 min. One unit of catalase activity (UCAT) was considered as the amount of enzyme necessary to catalyze 1 μmol of H_2_O_2_ per minute. GPx activity was analyzed at 340 nm through a protocol described by Ahmad *et al* [87]. One unit of GPx activity indicates how much enzyme is required to neutralize H_2_O_2_ using NADPH [88].

### Protein oxidative damage

Carbonyl concentration was determined in order to assess protein oxidative damage by using the DNPH alkaline method [89] and adjusting the optimal volumes for its use in 96 wells plates. 20 μL of DNPH (10 mM in 0.5 M H_3_PO_4_) were added to 20 μL of sample protein. Samples were incubated for 10 min in the dark with constant agitation. Afterwards, 10 μL of NaOH (6M) were added and incubated in the dark for 10 min at room temperature. Absorbance was determined at 450 nm against a blank where the protein solution was substituted by an equal volume of buffer solution. Carbonyl content was calculated as (Abs_450_/E)/total protein content of sample, where E = extinction factor of 46.1.

### Statistical analysis

All data were tested for normality for univariate analysis, with the D'Agostino & Pearson omnibus normality test and for homoscedasticity with Levene tests. When data met normality and homoscedasticity assumptions, an analysis of variance (ANOVA) was performed followed by a Tukey´s post hoc test when needed. When normality was met but homoscedasticity was not, an ANOVA with Welch correction and a post hoc Games-Howell test were conducted. When neither criterion was met, then we used a Kruskall-Wallis test followed by Dunn’s post hoc test if needed. All data were tested for differences between sexes with either a t-test or Mann-Whitney U test. Significant differences between sexes were found only for the PHA index on P1. Thus, an ANCOVA (Sex × Season) with scaled mass index as a covariate was performed to compare the PHA index. The stress stimuli effect was analyzed with a t-test. Principal component analysis (PCA) was conducted using the software PAST version 2.17c. Only data from individuals that had all variables measured were included in the analysis. All 12 variables were included (BA, SOD, CAT, GPx, PHA, SMI, %H, carbonyls, dŦ_a,_ Ŵ_n,_ sex and reproductive state: rep) for baseline analyses. We used the mean value for the ambient variables, from the day previous to individual collection. For post stress analyses of 6 h and 12 h, we included (BA6/12, SOD6/12, CAT6/12, GPx6/12, %H6/12, Carbonyls6/12, sex, and rep. All data included in the PCA´s had a normal multivariate distribution (Mardia test).

Principal component analysis baseline analyses (D-PCA) were computed in a correlation matrix to determine which variables had more weight on the model and how the variables interacted with each other during periods 1 and 2 separately. Variables with coefficient values < 0.5 were considered not to significantly contribute to the model and were removed from the analysis. Afterwards, a between-group analysis (BG-PCA) was computed in the correlation matrix in order to highlight the difference between groups (seasons) by measuring the contribution (in terms of variance) of the variables in the differentiation of individuals between groups.

## Results

### Weather conditions

**Fig 1:**
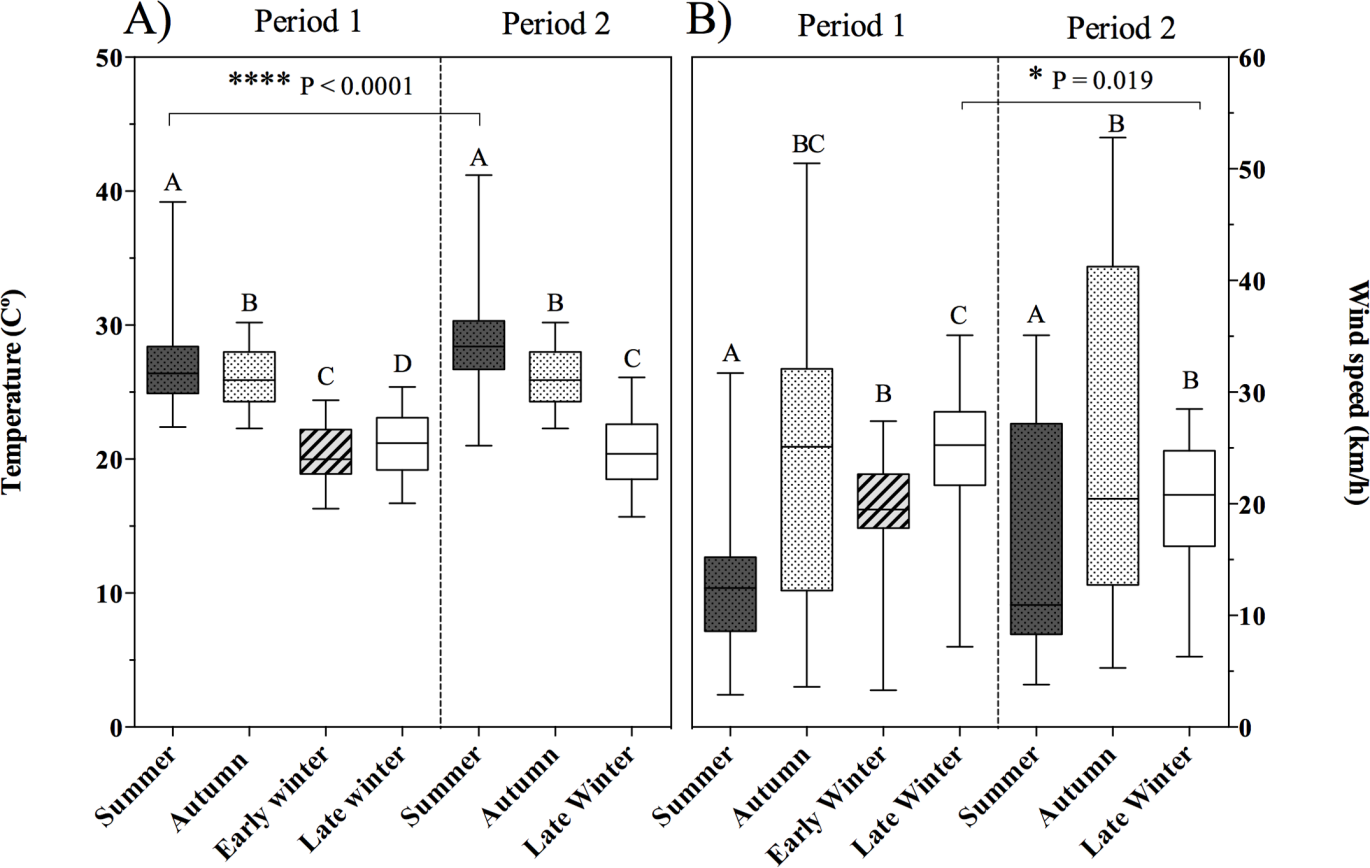
Weather conditions by season on both periods (period 1 and period 2) of sample collection. A) Temperature (ºC) (median ± interquartile range); B) Wind speed (km/h) (median ± interquartile range). Different letters indicate significant differences among seasons within a period and asterisks indicate significant differences between the same seasons across different periods, as determined by Kruskal-Wallis analysis of variance.

For period 1 (P1), daily mean ambient temperature (dŦ_a_) was different among seasons sampled (H_3_ = 1582, P < 0.0001). Summer had the highest dŦ_a_ (median, minimum-maximum: 26.4ºC, 22.4–39.2ºC) compared to autumn (25.9ºC, 22.3–30.2ºC; P = 0.0075), early winter (20.0º C, 16.3–24.4ºC; P < 0.0001) and late winter (21.2ºC, 16.7–25.4ºC; P < 0.0001). Autumn dŦ_a_ was higher than in early winter (P < 0.0001) and late winter (P < 0.0001), whereas dŦ_a_ from late winter was higher than dŦ_a_ from early winter (P = 0.003). For period 2 (P2), dŦ_a_ was also different among seasons (H_2_ = 1097, P < 0.0001). Summer (28.6ºC, 21.0–41.2ºC) was hotter than autumn (26.0ºC, 22.3–30.2ºC; P < 0.0001) and late winter (20.5ºC, 15.7–26.1ºC; P < 0.0001), and autumn was warmer than late winter (P < 0.0001) (Fig 1A). Both periods showed the same pattern, with the highest temperature in summer and the lowest in winter, but summer dŦ_a_ was significantly higher in P1 than P2 (H_6_ = 2453, P < 0.0001).

Median night wind speed (Ŵ_n_) was significantly different (H_3_ = 100.6, P < 0.0001) among P1 seasons: summer had the lowest Ŵ_n_ (12.4 km/h, 2.9–31.7 km/h) in comparison to autumn (25.1 km/h, 3.6–50.5 km/h; P < 0.0001), early winter (19.5 km/h, 3.3–27.4 km/h; P < 0.0001), and late winter (25.2 km/h, 7.2–35.1 km/h). Ŵ_n_ was higher at the end of winter than at the beginning (P = 0.0004). For period 2, there were also significant differences among seasons (H_2_ = 31.8, P < 0.0001). Ŵ_n_ was higher during autumn (25.9 km/h, 5.3–52.8 km/h; P < 0.0001) and late winter (20.2 km/h, 6.3–28.5 km/h; P = 0.001) than during summer (16.2 km/h, 3.8–35.0 km/h) (Fig 1B). Both periods showed the same kind of outline (H_6_ = 123.9, P < 0.0001), but Ŵ_n_ in late winter was significantly lower in P2 than in P1 (P < 0.019) (Fig 1B).

### Body condition

**Fig 2:**
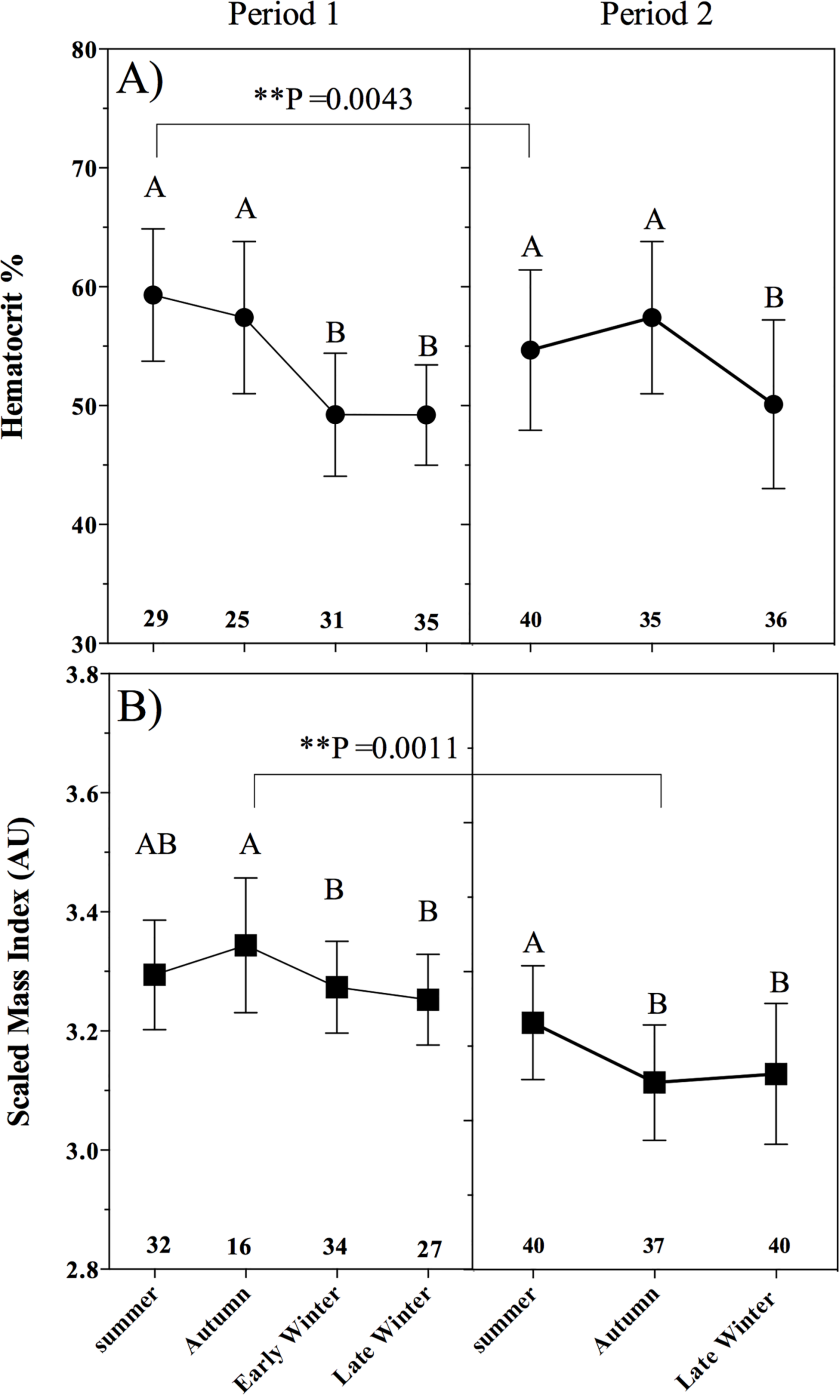
Bats body condition by season on both periods (period 1 and period 2) of sample collection. A) Hematocrit percentage (mean ± SD); B) Scaled mass index (mean ± SD). Different letters indicate significant differences between seasons within a period and asterisks indicate significant differences between the same seasons of different periods as determined by ANOVA. Numbers in bold indicate sample sizes.

Hematocrit varied across seasons during P1 (F_3, 116_ = 27.7, P < 0.0001), with higher values during summer and autumn than in early winter (P < 0.0001) and late winter (P < 0.0001). Hematocrit also varied in P2 (F_2, 108_ = 10.6, P < 0.0001) with lower hematocrit in late winter than in summer (P < 0.011) and autumn (P < 0.0001). There were significant differences in hematocrit between periods (F_6, 222_ = 15.9, P < 0.0001) but only for summer with a higher value in P1 (P = 0.004) (Fig 2A).

The scaled mass index (SMI) varied significantly among seasons during P1 (F_3, 104_ = 3.9, P = 0.010): it was significantly higher in autumn that in early winter (P = 0.045) and late winter (P = 0.007), but not significantly different from summer (P = 0.261). No significant differences were found in SMI between summer and early winter (P = 0.774) or late winter (P = 0.272). There were significant differences among seasons in P2 (F_2, 114_ = 10.6, P < 0.0001) but in a contrasting pattern; the SMI was lower in autumn than in summer (P = 0.0001) and late winter (P = 0.001). The SMI varied significantly between periods (F_6, 218_ = 5.47, P < 0.0001) but only in autumn during which the index was higher in P1 (P = 0.007) (Fig 2B).

### Seasonal enzyme antioxidant activity and protein damage

**Fig 3:**
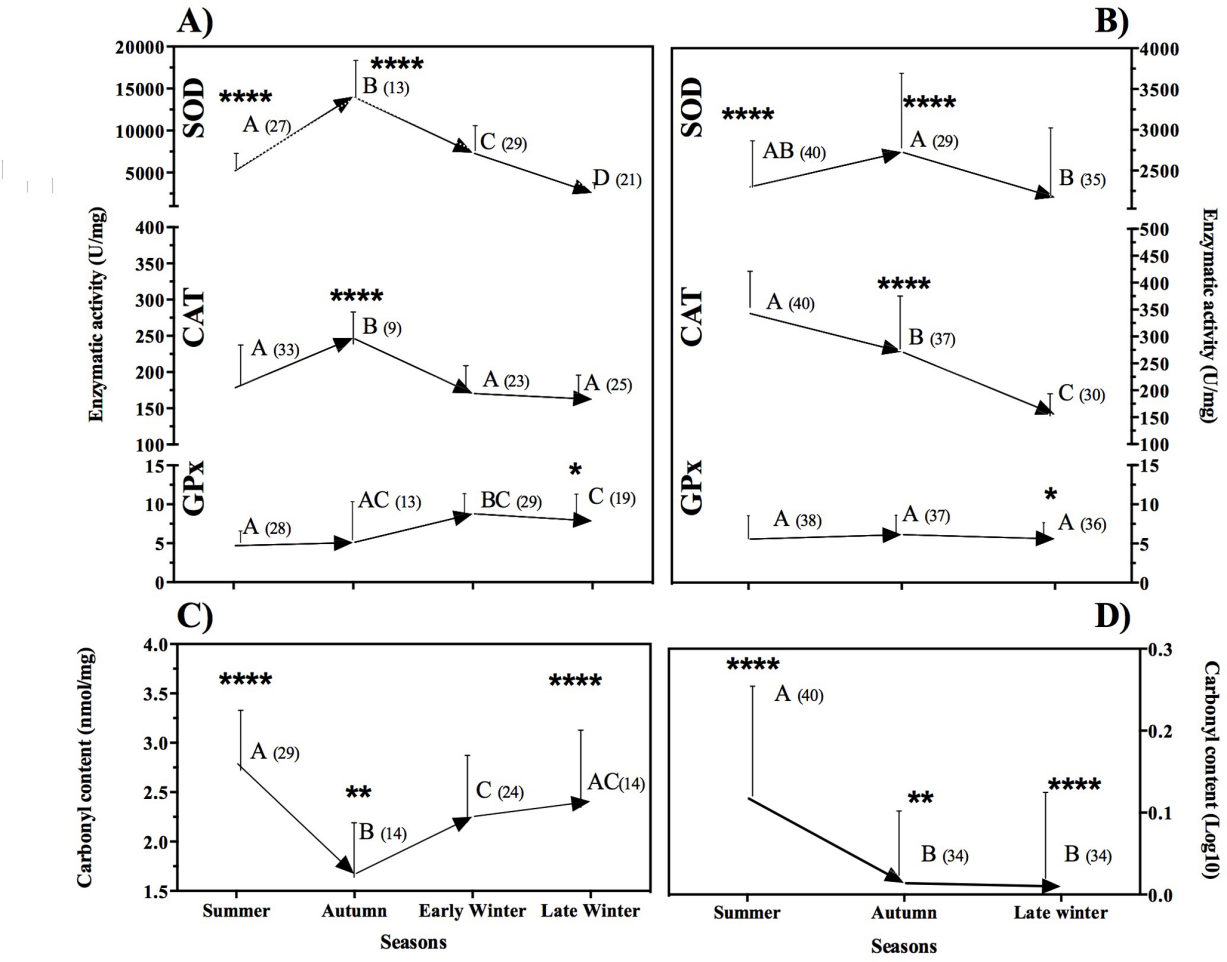
Oxidative stress on bats by season for both periods (period 1 and period 2) of sample collection. A) P1 antioxidant enzymatic activity in erythrocytes (mean ± SD). B) P2 antioxidant enzymatic activity in erythrocytes (mean ± SD). C) Protein damage measured by carbonyl quantification in erythrocytes (mean ± SD) on P1. D) Protein damage measured by carbonyl quantification in erythrocytes (mean ± SD) on P2. Different letters indicate significant differences among seasons of a period as revealed by ANOVA with Welch correction. Numbers in parenthesis indicate sample sizes.

Basal SOD activity varied significantly across seasons during P1 (F_3, 34.9_ = 51.2, P < 0.0001), with higher values in autumn than in early winter (P < 0.0001), late winter (P = 0.011) and summer (P = 0.001); in turn, basal activity during early winter was higher than in summer (P < 0.0001) and late winter (P < 0.0001), while activity during summer was higher than in late winter (P < 0.0001) (Fig 3A). There were significant differences among seasons in P2 (F_2, 58.4_ = 3.7, P = 0.030), with higher values in autumn than in late winter (P = 0.028) and a trend towards a higher value than in summer (P = 0.061), and no significant difference between summer and late winter (P = 0.716) (Fig 3B). Basal SOD activity during summer (P < 0.0001), and autumn (P < 0.0001), was higher in P1 than in P2 (F_6, 65.7_ = 33.4, P < 0.0001), and no other significant difference was found between periods for other seasons (Fig 3A and 3B).

CAT basal activity varied significantly among seasons in P1 (F_3, 29.2_ = 7.4, P = 0.001) with higher values in autumn than summer (P = 0.028), early winter (P = 0.11), and late winter (P = 0.004). No difference in CAT activity was found between summer and early winter (P = 0.97) and late winter (P = 0.48) (Fig 3A). CAT activity varied significantly among seasons in P2 (F_2, 66.4_ = 124.95, P < 0.0001) but with a contrasting pattern: activity was higher in summer than autumn: (P = 0.001) and late winter (P < 0.0001), and higher in autumn than late winter (P < 0.0001) (Fig 3B). Differences between periods (F_1, 61.8_ = 52.1, P < 0.0001) in CAT baseline levels were significant only in autumn when P2 had a higher value (P < 0.0001) (Fig 3A and 3B).

GPx basal activity varied significantly among seasons in P1 (F_3, 30.9_ = 14.8, P < 0.0001): activity was higher in early winter than in summer (P < 0.001) and autumn (P = 0.001), and not significant difference was found in GPx activity between early winter and late winter (P = 0.10) (Fig 3A). In contrast, no significant differences among seasons were found in P2 (F_2, 108_ = 0.7, P = 0.49) (Fig 3B). No significant differences were found between periods for any season (F_6, 64.2_ = 7.6, P <0.0001; autumn P1vs P2 P = 0.991; summer P = 0.703; late winter P = 0.915) (Fig 3A and 3B).

Protein damage varied significantly among seasons in P1 (F_3,77_ = 9.1, P < 0.0001): the highest protein damage was found in summer and late winter, with no significant difference between these seasons (P = 0.154), but both were significantly higher than in autumn (summer vs. autumn: P < 0.0001; late winter vs. autumn: P = 0.023). The damage in early winter was significantly higher than in autumn (P = 0.037), and significantly lower than in summer (P = 0.023), but no different than in late winter (P = 0.49) (Fig 3C). Protein damage varied significantly among seasons in P2 (F_2, 66.38_ = 6.4, P = 0.003) with significantly higher values in summer than in autumn (P = 0.003) and late winter (P = 0.009), and no difference between autumn and late winter (P = 0.999) (Fig 3D). Protein damage differed significantly between periods (H_7, 182_ = 119.1, P < 0.0001) with higher values in P1 for all seasons (P ≤ 0.005) (Fig 3C and 3D).

### Seasonal PHA challenge response

**Fig 4:**
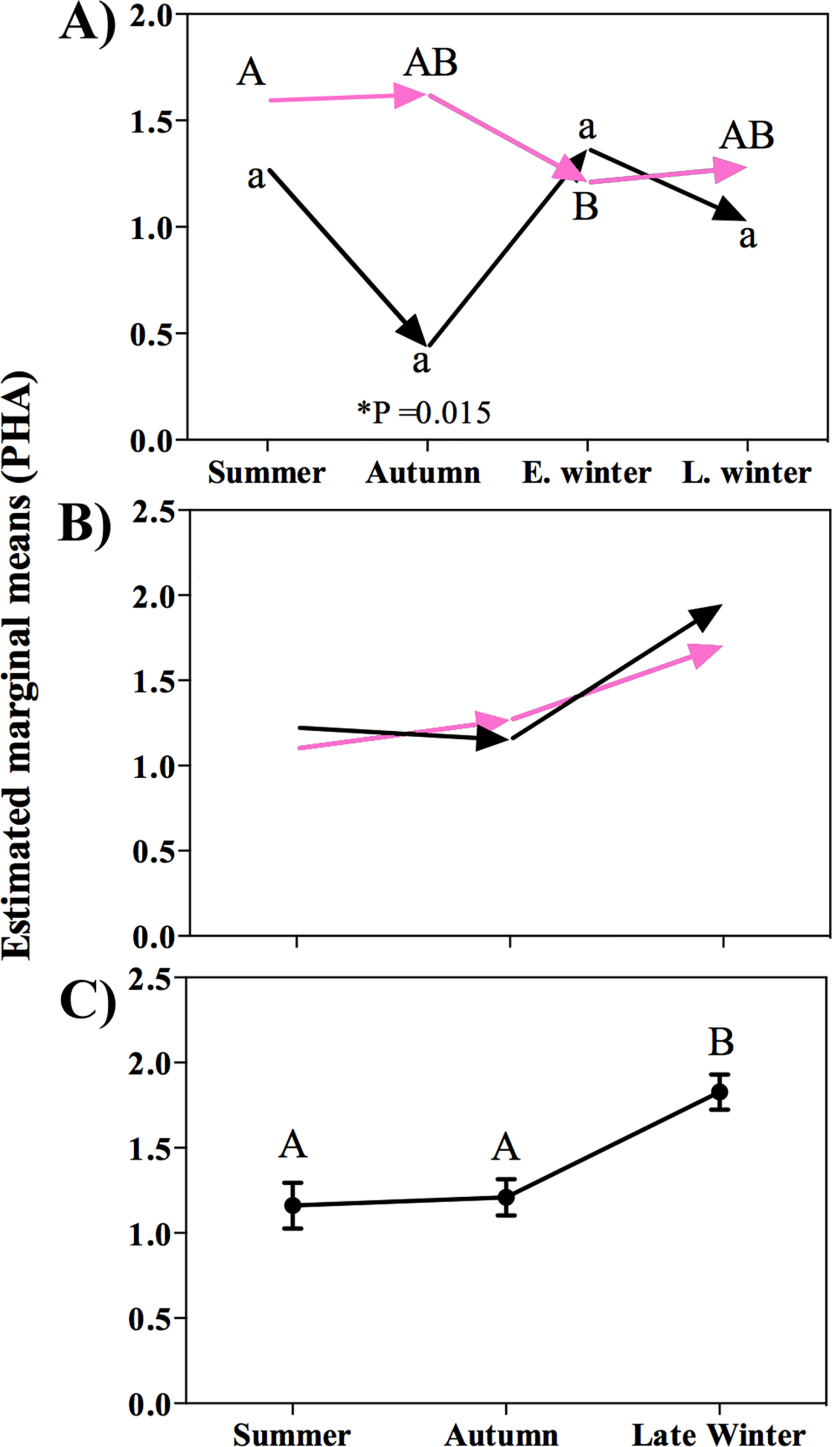
Seasonal swelling response after 6 hours of PHA post injection. A) P1, pink arrows represent seasonal variation in the swelling response in females; black arrows represent seasonal changes in the swelling response in males; different letters indicate significant differences among seasons within a period; asterisks indicate differences between periods for a given season. B) P2, pink arrows represent females and black arrows represent males; no significant differences were found. C) Combined data from P2 (estimated mean ± SE).

The ANCOVA model for PHA index after 6 h of the injection was significant on P1 (F_8, 96_ = 2.8, P = 0.009). After correcting for individual body conditions, the PHA response was significantly greater in females than males (F_1, 96_ = 8.3, P = 0.005). The magnitude of the PHA response did not differ significantly among seasons (F_3, 96_ = 1.5, P = 0.23) but the interaction between sex and season was significant (F_3, 96_ = 3.1, P = 0.029): females exhibited a more robust swelling response in summer than in early winter (P = 0.046) (Fig 4A).

**Fig 5:**
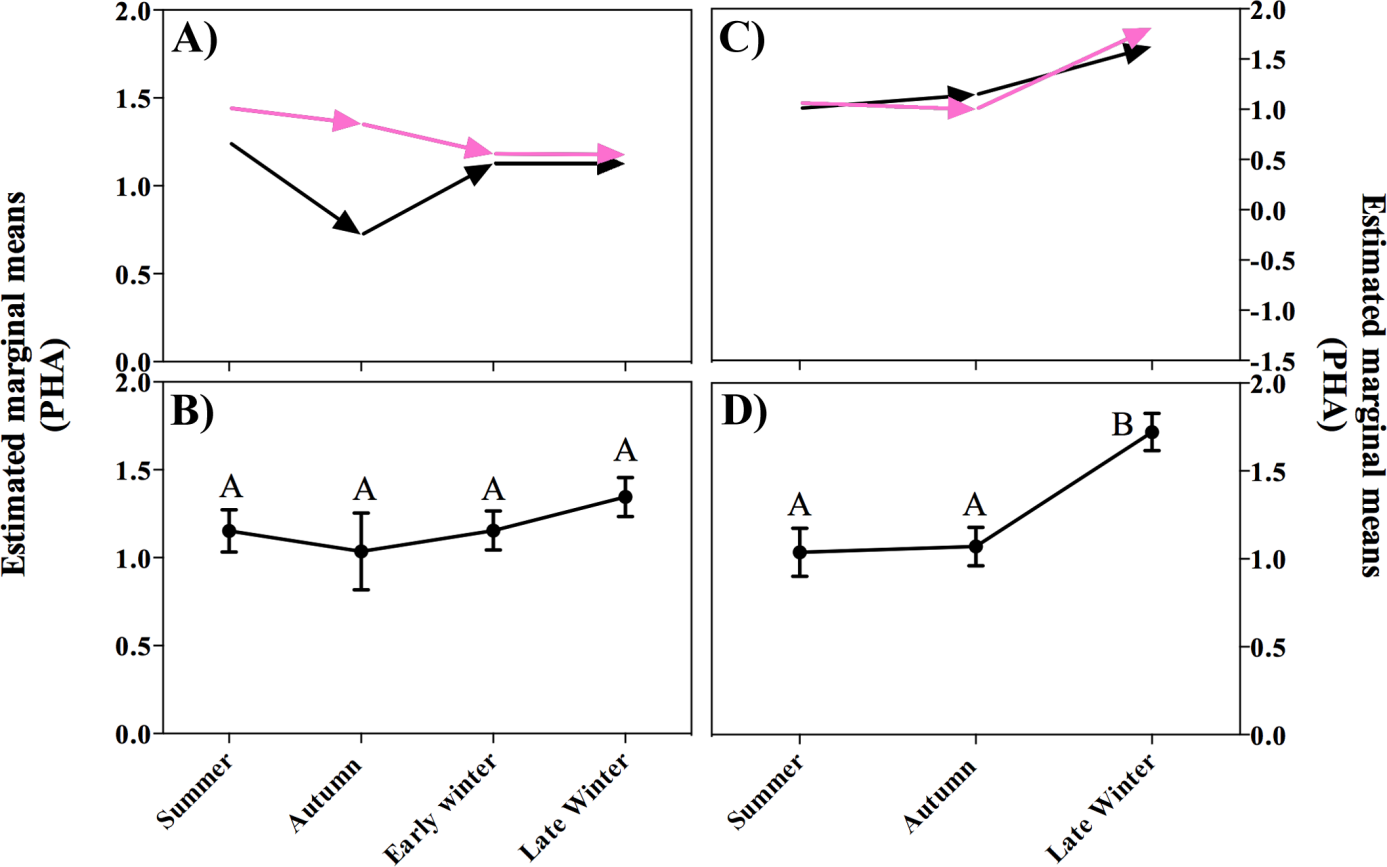
Seasonal swelling response after 12 hours of PHA injection. A) Data separated by sex from P1. B) Combined data from P1 (estimated marginal mean ± SE). C) Data separated by sex from P2. Pink arrows represent seasonal variation in the swelling response in females; black arrows represent seasonal changes in swelling response in males. D) Combined data from P2 (estimated marginal mean ± SE); different letters indicate significant differences among seasons.

The ANCOVA model for PHA index after 12 hours of the injection on P1 was not significant (F_8, 96_ = 0.68, P = 0.71); neither sex (F_1, 94_ = 2.5, P = 0.12) (Fig 5A), nor season (F_3, 94_ = 0.9, P = 0.46) (Fig 5B) had a significant effect on PHA swelling response. The interaction between sex and seasons was also non-significant (F_3, 94_ = 0.5, P = 0.66).

**Fig 6:**
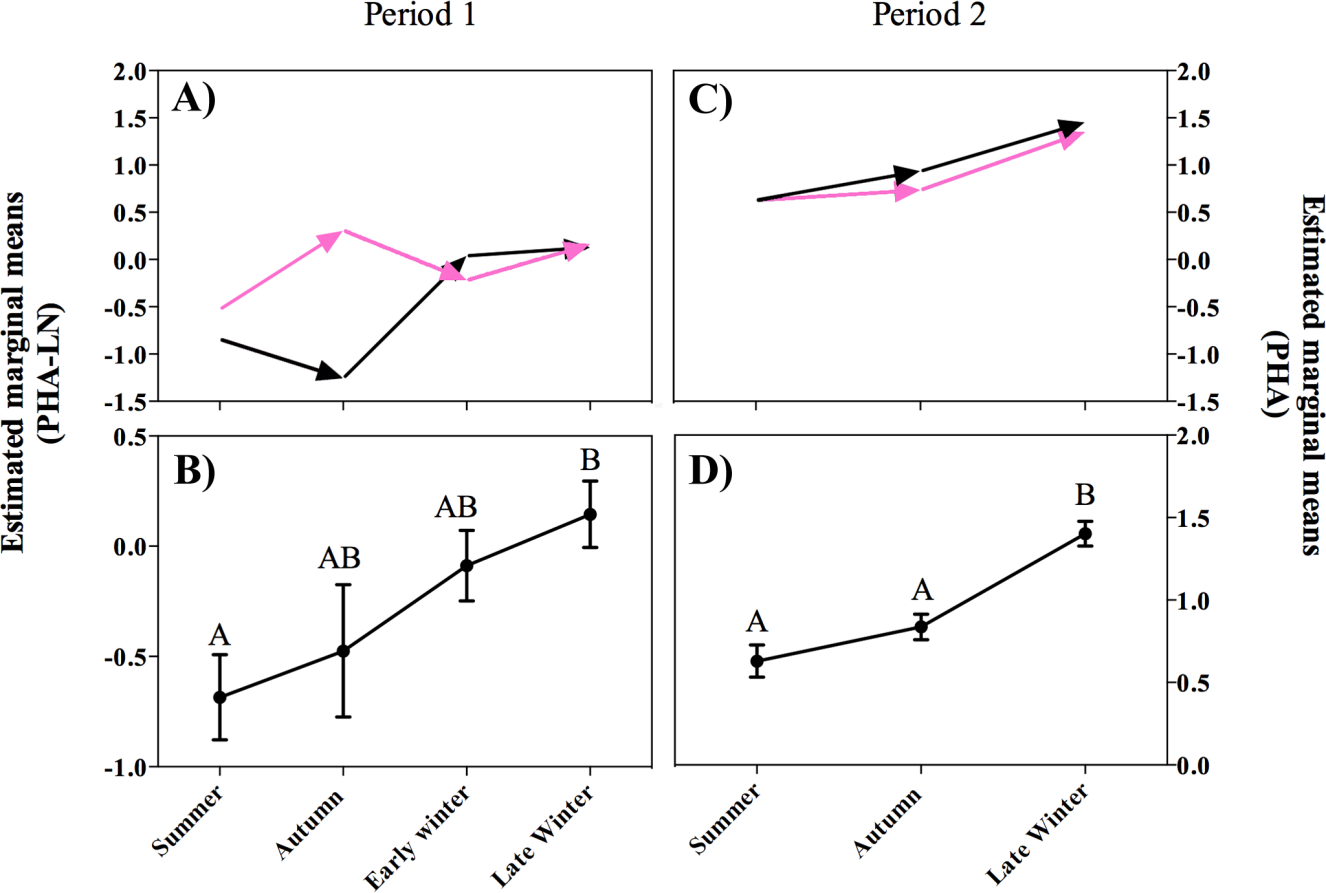
Seasonal swelling response after 24 hours of PHA injection. A) Data separated by sex from P1. B) Combined data from P1 (estimated marginal mean ± SE). C) Data separated by sex from P2. Pink arrows represent seasonal variation in the swelling response in females; black arrows represent seasonal changes in swelling response in males; D) Combined data from P2 (estimated marginal mean ± SE); different letters indicate significant differences among seasons.

The ANCOVA model for PHA after 24 hours was significant (F_8, 94_ = 2.7, P = 0.01). The effect of body condition was not significant (F_1, 94_ = 3.9, P = 0.758), and after correcting for body condition the PHA swelling response was not significantly different between sexes (F_1, 94_ = 3.9, P = 0.051) (Fig 6A), but it did differ significantly among seasons (F_3, 94_ = 4.1, P = 0.009) (Fig 6B). The magnitude of the swelling response in late winter was higher than in summer (P = 0.007), but was not significantly different than in autumn (P = 0.14) or early winter (P = 1.0). The swelling response in summer was not different than in autumn (P = 0.14) or early winter (P = 1.0).

The ANCOVA model for P2 after 6 h injection was also significant (F_6, 105_ = 5.3, P <0.0001). After correcting for body conditions of individuals, the magnitude of the PHA response was not significantly different between sexes (F_1, 105_ = 0.4, P = 0.553) (Fig 4B), but was different among seasons (F_2, 105_ = 11.9, P <0.0001) (Fig 4C). The interaction between sex and season was not significant (F_2, 105_ = 0.8, P = 0.452). The model for the PHA index after 12 hours was significant (F_6, 106_ = 5.9, P <0.001), but after correcting for the effect of body condition, significant differences were found among seasons (F_2, 106_ = 12.3, P <0.001) (Fig 5D) but not between sexes (F_1, 106_ = 0.5, P = 0.83) (Fig 5C). The magnitude of the swelling after 12 h of injection was greater in late winter than in autumn (P <0.0001) and summer (P = 0.001). The interaction between sex and season was not significant (F_2, 106_ = 0.7, P = 0.52). The model describing the magnitude of the PHA response after 24 hours was significant (F_6, 97_ = 9.9, P <0.0001). The effect of body condition was significant (F_1, 97_ = 4.1, P = 0.047).After correcting for the effect of body condition, we found no significant difference between sexes (F_1, 97_ = 1.1, P = 0.307) (Fig 6C), but swelling was significantly different among seasons (F_2, 97_ = 24.1, P <0.0001) (Fig 6D). Similar to P1, swelling response was higher in late winter than summer (P <0.001) and autumn (P <0.001). The interaction between sex and season was not significant (F_2, 97_ = 0.32, P = 0.715).

### Seasonal Bactericidal Activity (BA)

**Fig 7:**
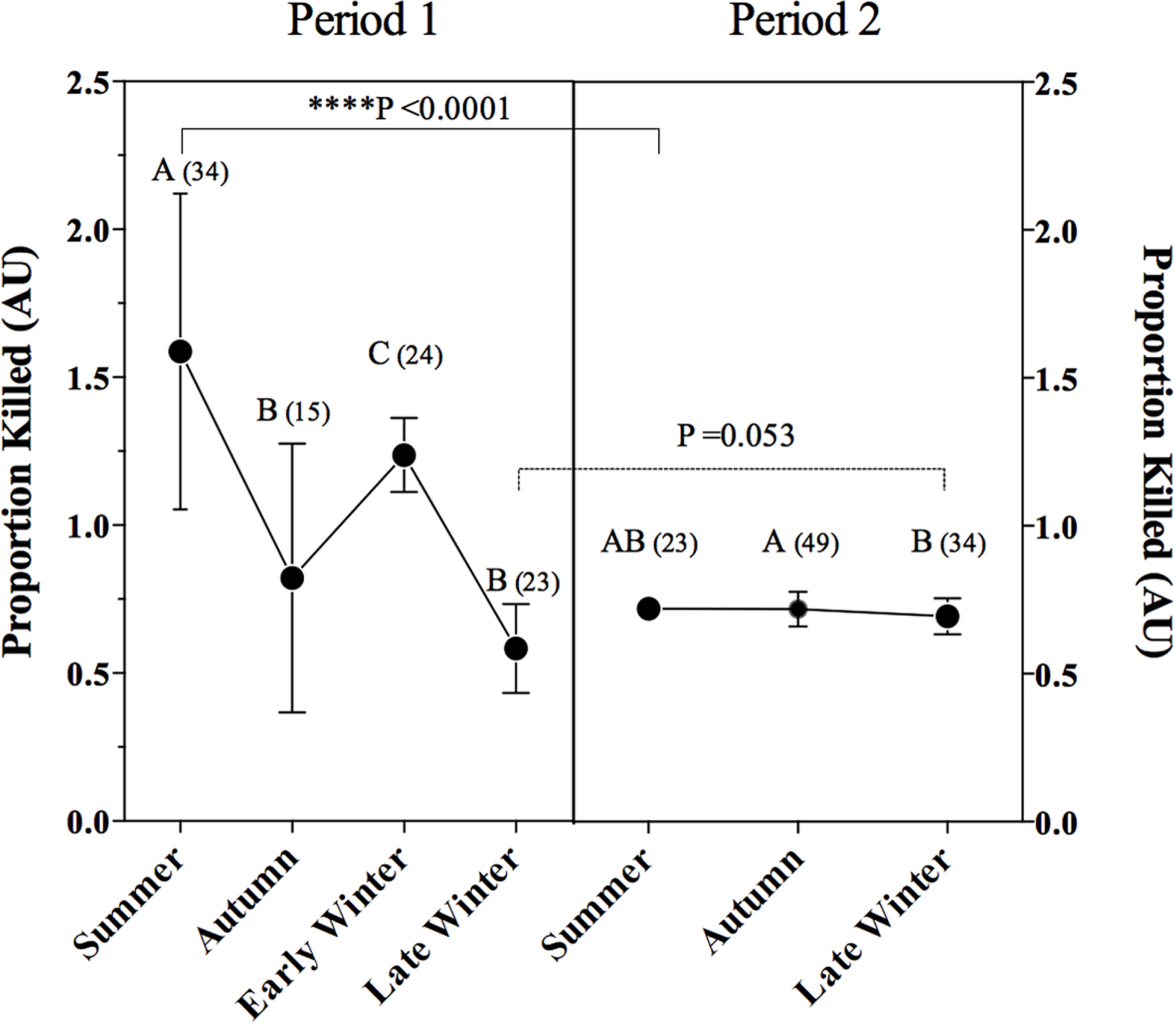
Bactericidal activity of plasma. Samples correspond to seasons within periods 1 (mean ± SD), and 2 (median ± interquartile range), as revealed by ANOVA analysis with Welch correction and Kruskal-wallis analysis, respectively. Different letters indicate significant differences between seasons within a period, asteisks indicate significant differences between the same season of different periods, as revealed by Kruskal-Wallis post-hoc analysis. Numbers in parenthesis indicate sample sizes.

BA of plasma samples collected immediately after capture was different among seasons in P1 (F_3, 41.13_ = 55.14, P < 0.0001), with higher values in summer than autumn (P < 0.0001), early winter (P = 0.003), and late winter (P < 0.0001). Plasma from bats collected in early winter had higher BA than in late winter (P = 0.047) and autumn (P < 0.0001) (Fig 7) BA varied significantly in P2 (H_2, 94_ = 6.3, P = 0.043) with higher values in autumn than late winter (P = 0.046), but similar to levels in the summer (P > 0.999). BA varied between periods (H_6, 185_ = 120.3, P < 0.0001) but only in summer with higher values in P1 (P < 0.0001).

### Effect of acute stress stimuli

**Fig 8:**
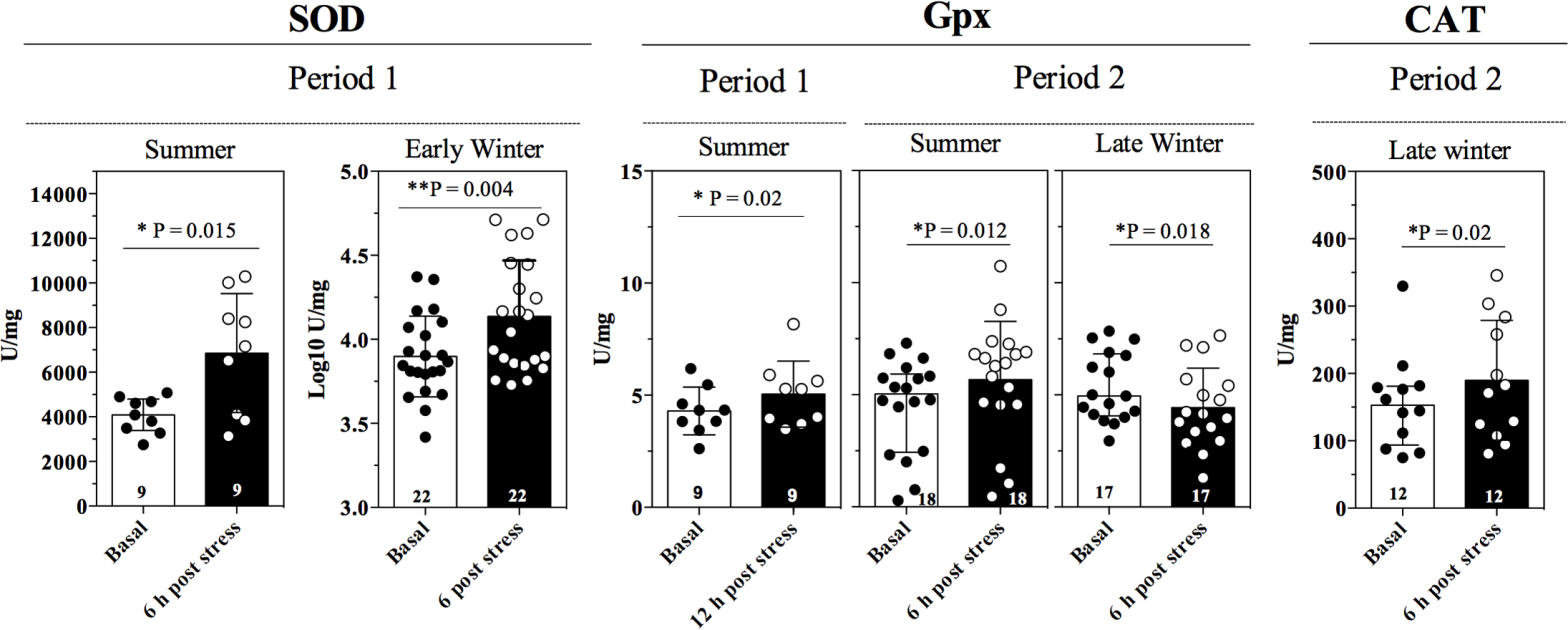
Acute stress stimuli impact on physiological markers. Change in SOD, CAT and GPx activity (mean ± SD). Asterisks indicate significant differences in the response between basal and post-stress levels based on Student’s t-tests. Bold numbers inside bars indicate sample sizes.

**Fig 9:**
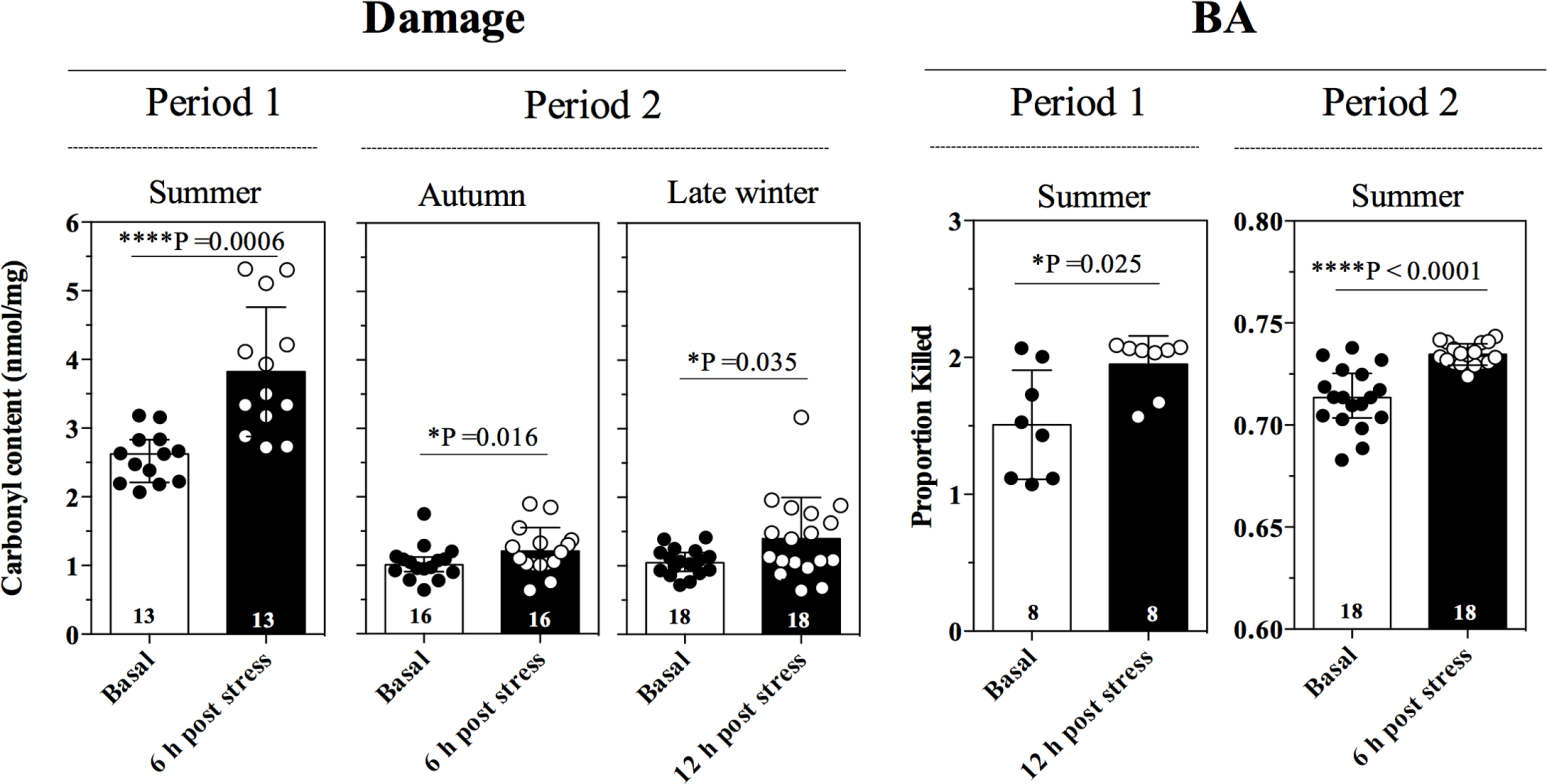
Acute stress stimuli impact on physiological markers. Change in carbonyl content and BA (mean ± SD) Asterisks indicate significant differences in the response between basal and post-stress levels based on Student’s t-tests. Bold numbers inside bars indicate sample sizes.

Compared to basal levels, SOD activity was greater after 6 h of immobilization stress during summer (t_8_ = 3.06, P = 0.015) and during early winter (t_21_ = 3.19, P = 0.0044) and GPx activity was increased after 12 h of immobilization during summer (t_8_ = 2.88, P = 0.021; Fig 8) in P1. No increase in carbonyls was detected in early winter (t_7_ = 1.53, P = 0.17); however, carbonyl content in summer after 6 h of stress during the summer (t_7_ = 2.82, P = 0.025) BA increased during summer after 12 h of stress (t_17_ = 2.82, P = 0.025; Fig 9). In P2, CAT activity was greater in late winter (t_11_ = 2.696, P = 0.021) and GPx activity was greater in summer (t_17_ = 2.830, P = 0.012) and lower in late winter (t_16_ = 2.629, P = 0.018; Fig 8) compared to basal levels. Carbonyl content did not increase following immobilization in summer, but it increased in autumn after 6 h of stress (t_15_ = 2.701, P = 0.016) and in late winter after 12 h of stress (t_17_ = 2.285, P = 0.035; Fig 9). BA increased during summer after 6 h of stress (t_17_ = 5.47, P <0.0001; Fig 9).

### Principal component analysis (PCA): Inter-correlation between variables

**Fig 10:**
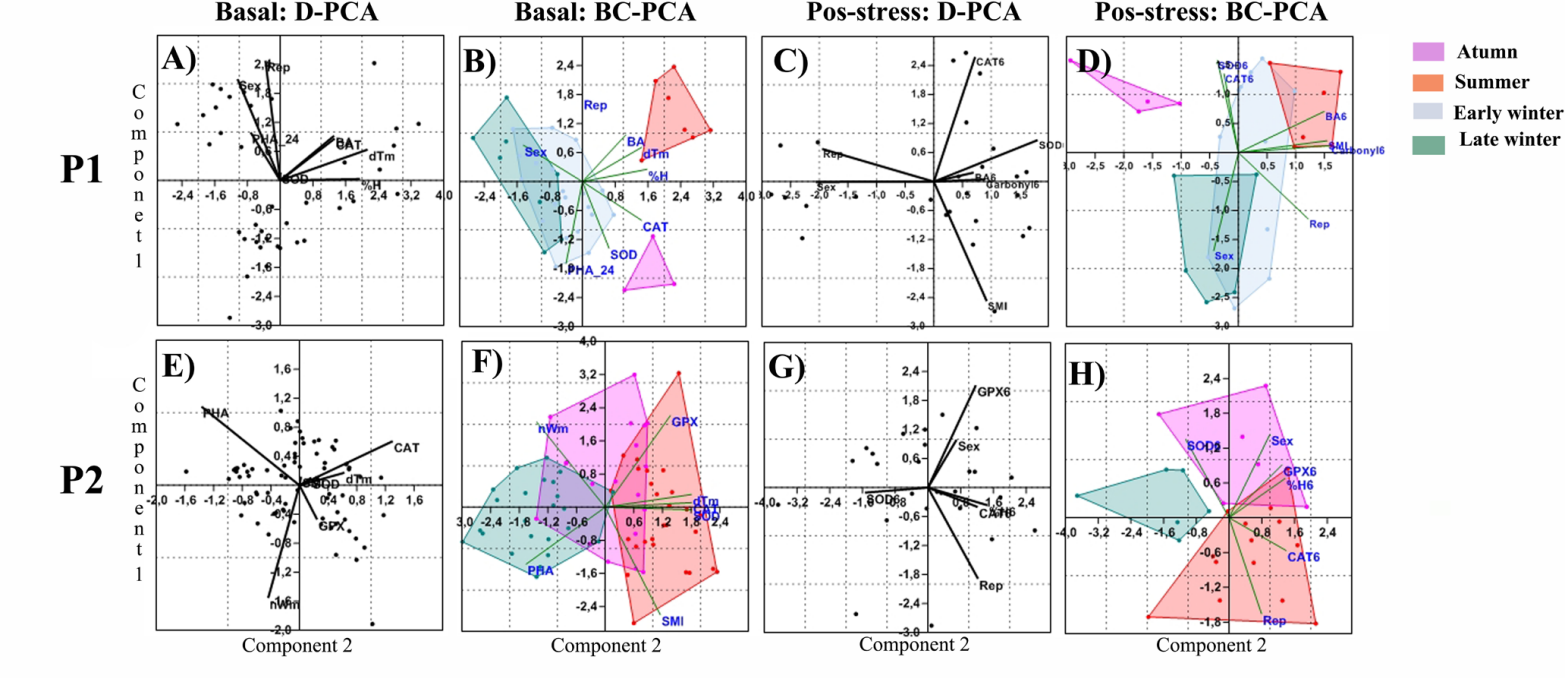
Graphic representation of principal component analysis plotting PC1 against PC2. A) Period 1 basal disregard-PCA, B) Period 1 basal between groups-PCA, C) Period 1 six hours Post-stress disregard-PCA, D) Period 1 six hours Post-stress between groups-PCA, E) Period 2 basal disregard-PCA, F) Period 2 basal between groups-PCA, G) Period 2 six hours Post-stress disregard-PCA, H) Period 2 six hours Post-stress between groups-PCA.

The PCA made on the basal values of the physiological markers, the first four components explained 83% of the variance, independent of groups (D-PCA) in P1. PC1 was correlated with dŦ_a_ (+) and %H (+), PC2 with sex (+) and reproductive status (rep) (+), PC3 with SOD (+), and PC4 with CAT (+), PHA (+) and BA (-) (Table 1). When plotting PC1 against PC2 (Fig 10A), bats had higher CAT and SOD activity levels, and higher hematocrit percentage and BA at high T_a_. When T_a_ was low, bats had higher swelling responses, but in particular reproductive males presented the highest PHA index. The between-group principal component analysis (BG-PCA) explained 92% of the variance within the first two components, which pointed out the variables responsible of separating the groups in PC1 (+CAT, +H%, +dŦ_a_, +sex) and PC2 (+SOD, +PHA, +rep) (Table 1). The biplot (Fig 10B) revealed a clear separation among groups, except for early and late winter, which displayed a spliced area. T_a_ was at its highest level in summer and bats were mainly characterized for having high BA and H% during this period. Bats had their highest SOD and CAT activity in autumn, while during early winter they still retained some CAT activity. All bats in late winter had low SOD and CAT activity but high PHA response, particularly male bats. Although low temperatures were related with low AEA, protein oxidative damage seemed not to play an important role (coefficient lower than 0.5) in the model of undisturbed bats. The D-PCA of post stress data (6 hours) (Table 1) explained 78% of variance within the first four components (Table 1). PC1 was correlated with SOD6 (+), sex (-) and rep (-), PC2 with CAT6 (+) and SMI (-), PC3 with BA6 (+), and PC4 with carbonyl6 (-) (Table 2, supplementary material). Stress response was highly dependent of bat sex and reproductive state, with non-reproductive females having higher SOD and CAT activity, as well as an increment in BA and carbonyls but not as strong, suggesting that in general they are capable of mounting an efficient defense against unknown sources of stress, especially when they have low body conditions (Fig 10C). The post stress data (6 hours) BG-PCA explained 91% of the variance within the first two components. PC1 (+Carbonyl6, +BA6, +SMI, +rep) suggest that reproductive bats with good body condition had higher protein damage and cellular immune response, and PC2 (+SOD6, +CAT6, -sex) indicates that females had higher antioxidant defenses (Table 1). The biplot (Fig 10D) reveals an even further group separation compared to the basal data BG-PCA. During autumn and summer, bats mounted a high CAT and SOD response, but during summer bats had increased BA and protein damage. The area plot for early winter expanded, so it overlapped with late winter and with summer; this divergent stress response was sex dependent, with females being more prone to have high antioxidant defenses and high protein damage in contrast to males.

**Table 1:** Component correlation matrix of principal component analysis (PCA) used to determine relationships between physiological variables in the first period of sample collection.

Type	PCA	Variable	PC1	PC2	PC3	PC4
Basal	D	Eigenvalues	234.605	187.294	143.655	0.990458
		Percent of variance	29.33	23.41	17.96	12.38
		Cumulative %	29.33	52.74	70.70	**83.08**
		BA	0.357	0.251	0.307	**-0.533**
		SOD	0.001	0.045	**0.736**	-0.271
		CAT	0.361	0.235	0.284	**0.515**
		PHA_24	-0.196	0.270	0.357	**0.580**
		%H	**0.529**	0.009	-0.214	0.120
		Sex	-0.283	**0.569**	-0.207	-0.078
		Rep	-0.098	**0.673**	-0.181	-0.140
		dTm	**0.579**	0.174	-0.184	0.033
	BG	Eigenvalues	397.53	338.96		
		Percent of variance	49.69	42.37		
		Cumulative %	49.691	**92.061**		
		BA	0.327	0.299		
		SOD	0.201	**-0.426**		
		CAT	**0.446**	-0.247		
		PHA_24	-0.125	**-0.524**		
		%H	**0.488**	0.079		
		Sex	**-0.447**	0.233		
		Rep	-0.001	**0.533**		
		dTm	**3.000**	0.220		
6h stress stimuli	D	Eigenvalues	22.64	170.31	125.01	100.37
		Percent of variance	28.30	21.29	15.63	12.55
		Cumulative %	28.30	49.59	65.21	**77.76**
		BA6	0.1855	0.05007	**0.692**	0.5319
		SOD6	**0.4847**	0.23	-0.043	0.4012
		CAT6	0.1922	**0.6893**	0.1077	-0.2285
		Carbonyl 6	0.2393	0.01307	0.4773	**-0.6999**
		SMI	0.2466	**-0.6607**	0.2744	-0.0659
		Sex	**-0.5508**	-0.001836	0.1636	0.09569
		Rep	**-0.5218**	0.181	0.4217	0.02447
	BG	Eigenvalues	402.16	312.10		
		Percent of variance	50.27	39.01		
		Cumulative %	50.27	**89.28**		
		BA6	**0.4965**	0.2353		
		SOD6	-0.1217	**0.5271**		
		CAT6	-0.08278	**0.4504**		
		Carbonyl6	**0.5327**	0.04164		
		SMI	**0.5142**	0.06826		
		Sex	-0.1432	**-0.5606**		
		Rep	**0.404**	-0.3786		

Note. PCA analysis was performed as Disregard PCA analysis (D), and between groups PCA analysis (BG). Loadings highlighted in bold and underlined represent the highest absolute value of the variable when considered different PCs. BA = Bactericidal activity, BA6 = BA after 6 h stress, SOD = Super oxide dismutase, SOD6 = SOD after 6 h stress, CAT = catalase, CAT6 = CAT after 6 h stress, Gpx = Glutathione peroxidase, GPx6 = GPx after 6 h stress, %H = percent of hematocrit, rep = reproductive state, SMI = scaled mass index, Carbonyl6 = carbonyl content after 6 h stress, dTm = mean daily temperature, PHA_24 = Phytohaemagglutinin 24 h index.

**Table 2:** Component correlation matrix of principal component analysis (PCA) used to determine relationships between physiological variables in the second period of sample collection.

Type	PCA	Variable	PC1	PC2	PC3	PC4
Basal	D	Eigenvalues	210.88	15.10	107.92	0.97
		Percent of variance	30.13	21.57	15.42	13.87
		Cumulative %	30.13	51.70	67.12	**80.98**
		SOD	-0.018	0.350	**0.761**	0.267
		CAT	**0.561**	0.161	0.089	0.309
		GPX	0.164	0.305	-0.584	**0.598**
		PHA	-0.361	-0.308	0.188	**0.643**
		SMI	0.311	**-0.536**	0.115	0.191
		dTm	**0.607**	0.118	0.151	-0.160
		nWm	-0.250	**0.602**	-0.001	-0.011
	BG	Eigenvalues	541.89	** **		
		Percent of variance	77.41			
		Cumulative %	**77.41**			
		SOD	**0.430**	-0.015		
		CAT	**0.429**	0.026		
		GPX	0.323	0.524		
		PHA	**-0.392**	-0.325		
		SMI	0.273	-0.614		
		dTm	**0.428**	0.073		
		nWm	-0.340	0.487		
6h stress stimuli	D	Eigenvalues	195.15	109.88	0.98	0.91
		Percent of variance	32.53	18.31	16.40	15.16
		Cumulative %	32.53	50.84	67.24	**82.41**
		BA6	0.186	0.050	**0.692**	0.532
		SOD6	**0.485**	0.230	-0.043	0.401
		CAT6	0.192	**0.689**	0.108	-0.229
		Carbonyl 6	0.239	0.013	0.477	**-0.700**
		SMI	0.247	**-0.661**	0.274	-0.066
		Sex	**-0.551**	-0.002	0.164	0.096
		Rep	**-0.522**	0.181	0.422	0.024
	BG	Eigenvalues	388.20	211.80		
		Percent of variance	64.70	35.30		
		Cumulative %	64.70	**100.00**		
		BA6	**0.497**	0.235		
		SOD6	-0.122	**0.527**		
		CAT6	-0.083	**0.450**		
		Carbonyl6	**0.533**	0.042		
		SMI	**0.514**	0.068		
		Sex	-0.143	**-0.561**		
		Rep	**0.404**	-0.379		
12h stress stimuli	D	Eigenvalues	219.90	163.34	119.06	107.31
		Percent of variance	27.49	20.42	14.88	13.41
		Cumulative %	27.49	47.90	62.79	**76.20**
		SOD 12	**-0.668**	0.374	0.491	0.098
		CAT 12	0.251	0.396	**0.764**	0.187
		GPX 12	-0.034	0.247	-0.277	**0.775**
		CARBONYL 12	**0.586**	0.240	0.062	-0.510
		SMI	**0.687**	0.319	0.018	-0.045
		H% 12	**0.592**	0.341	-0.343	0.235
		Sex	**-0.712**	0.407	-0.362	-0.307
		Rep	-0.124	**0.914**	-0.190	-0.132
		Eigenvalues	537.466	262.534		
		Percent of variance	67.183	32.817		
		Cumulative %	67.183	**100.000**		
		SOD 12	0.293	**0.453**		
		CAT 12	**0.392**	-0.258		
		GPX 12	0.163	**0.571**		
		CARBONYL 12	**0.402**	0.223		
		SMI	**0.431**	-0.014		
		H% 12	0.250	**-0.503**		
		Sex	**-0.375**	0.306		
		Rep	**0.428**	0.075		

Note. PCA analysis was performed as Disregard PCA analysis (D), and between groups PCA analysis (BG). Loadings highlighted in bold and underlined represent the highest absolute value of the variable when considered different PCs, BA6 = BA after 6 h stress, BA12 = BA after 12 h stress, SOD = Super oxide dismutase, SOD6 = SOD after 6 h stress, SOD12 = SOD after 12 h stress, CAT = catalase, CAT6 = CAT after 6 h stress, CAT12 = CAT after 12 h stress, Gpx = Glutathione peroxidase, GPx6 = GPx after 6 h stress, GPx12 = GPx after 12 h stress, %H = percent of hematocrit, %H12 = %H after 12 h stress, rep = reproductive state, SMI = scaled mass index, Carbonyl6 = carbonyl content after 6 h stress, dTm = median daily temperature, nWm = night median wind speed, PHA = Phytohaemagglutinin 24 h index.

**Fig 11:**
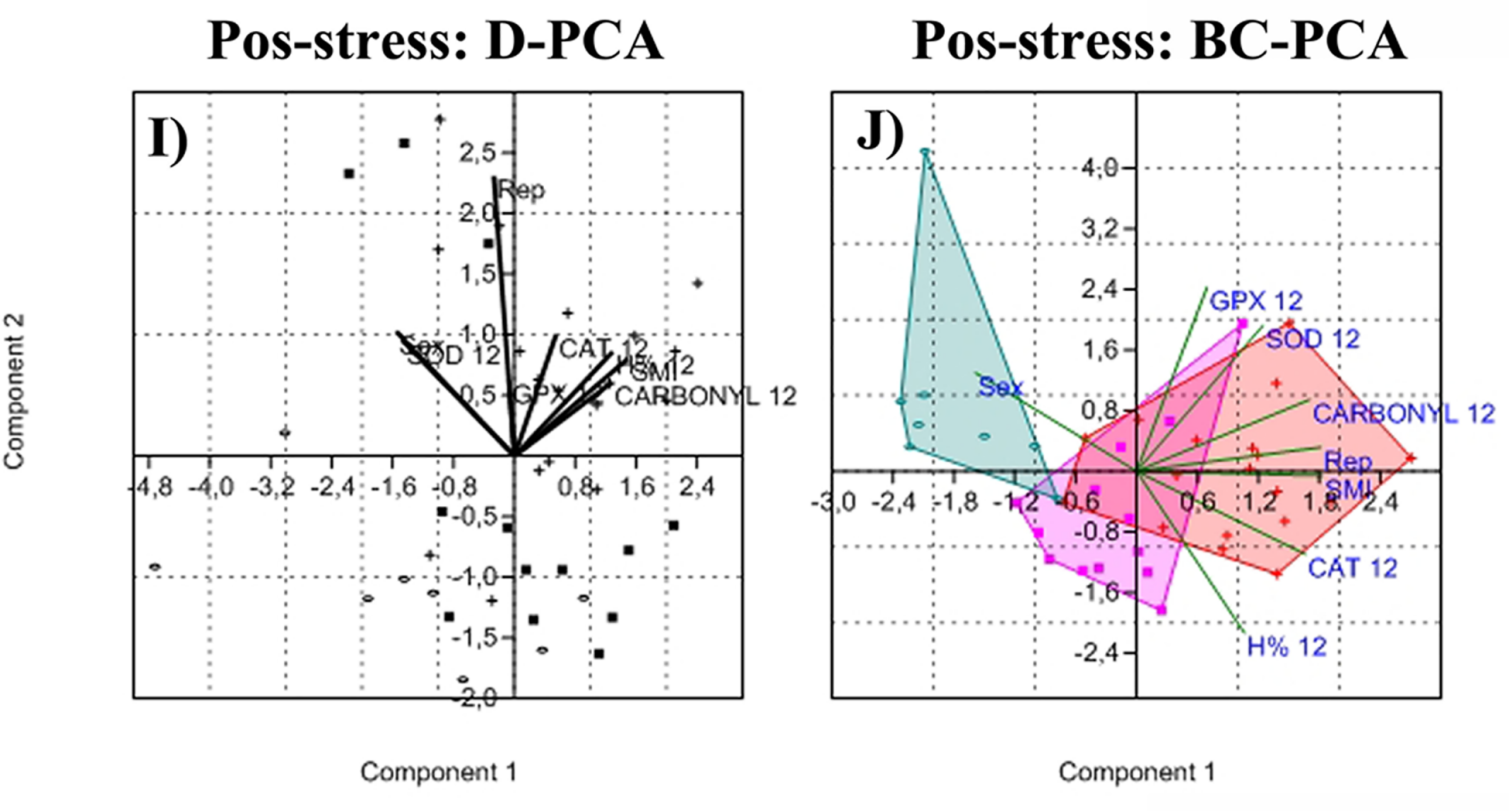
Graphic representation of principal component analysis, plotting PC1 against PC2. I) Period 2 twelve hours Post-stress disregard-PCA, H) Period 2 twelve hours Post-stress between groups-PCA.

Basal values explained 81% of the variance in the first four components of D-PCA in P2 (Table 2). PC1 was correlated with CAT (+) and dŦ (+), PC2 with Ŵ_n_ (+) and SMI (+), PC3 with SOD (+), and PC4 with GPx (+) and PHA (+) (Table 2). When plotting PC1 against PC2 (Fig 10E), bats had higher antioxidant activity levels (especially CAT) with high T_a_, while during windy nights GPx was the main antioxidant defense followed by SOD. The swelling response tended to be greater on low T_a_ and windy nights, and similarly to P1, protein oxidative damage did not to have an important effect during basal conditions. The BG-PCA explained 77% of the variance within the first component (Table 2), with SOD (+), CAT (+), dŦ (+), and PHA (-), as the variables mainly responsible for separating the groups. The biplot (Fig 10F) showed a separation between summer and winter, with autumn between these seasons. During summer, bats had high SOD, CAT and GPx activity and a greater SMI, whereas in winter bats had strong swelling responses and low SOD, CAT and GPx activity. 82% of the variation in the post stress (6 hours) response was explained by the first four components of the D-PCA data: PC1was correlated with H%6 (+), PC2 with GPx6 (+) and rep (-), PC3 with CAT (-) and sex (+), and PC4 with SOD (+) (Table 2). The biplot (Fig 10G) showed that non-reproductive males enhanced their GPx activity during the stress response, while lactating females enhanced their CAT activity, but bats that presented high SOD activity had low CAT or GPx activity. The post stress BG-PCA data explained 100% of the variance in PC1 (+CAT6, +GPx6, +%H6) and PC2 (+SOD6, -rep, +sex; Table 2). The biplot (Fig 10H) revealed that bats from each season had distinctly different stress responses. During autumn, non-reproductive males increased their SOD and CAT activity, while during summer bats (that in basal conditions had high SOD, CAT and GPx activity) only enhanced their CAT activity, particularly lactating females., Antioxidant activity increased in winter but a lesser extent compared to autumn and summer. As in P1, variation in protein oxidative damage did not correlate strongly with other parameters in the 6 hours post-stress model. 76% of variance in the post stress (12 hour) model was explained by the first four components of the D-PCA analysis: PC1 was correlated with SOD12 (-), Carbonyl 12 (+), SMI (+), H%12 (+) and sex (-), PC2 with rep (+), PC3 with CAT (+) and PC4 with GPx (+) (Table 2). The biplot (Fig 1) showed that after 12 h of stress, bats (especially lactating females and in good body condition) increased their AEA and protein carbonylation; however, there was a significant negative correlation between SOD and Carbonyls in PC1. The post stress BG-PCA data explained 100% of the variance in PC1 (+CAT12, +Carbonyl12, +SMI, +sex, +rep), and PC2 (+SOD12, +GPx12, +H%12) (Table 1). The biplot (Fig 11J) showed that after 12 h of stress, bats had a similar response in summer and autumn, in contrast to bats from winter. Lactating females with high SMI increased CAT, SOD and GPx activities and protein carbonylation in summer. Surprisingly, bats in winter had low CAT, SOD and GPX activities and a very low increase in carbonylation.

## Discussion

The study of physiological changes from basal to post-stress levels at different times of the year has largely been neglected; as a consequence, the few available published studies report contradictory results [9,54,55]. We propose that such discrepancies arise from a heterogeneous sampling method, and that just as basal levels set points are dynamic through the year, stress response also varies across the annual cycle. Therefore, animals would be more susceptible to unpredictable sources of stress at certain times of the year. We evaluated basal immunocompetence and redox state change during autumn, summer and winter and assessed the effect of acute stress. Our results showed that the basal immune response was an important physiological response through the year, although the particular type of response depended on the time of the year.

PHA response and plasma bactericidal activity are functionally different immune response measurements. Plasma BA is a constitutive response related to humoral resistance to extracellular infections, while the PHA response is an induced, mostly cellular, immune response related to resistance to intracellular infections [90]. However, recent debate has questioned the interpretation of PHA injection-site swelling as an index of T cell-mediated immunocompetence, and it has been reported that other leukocytes (basophils, neutrophils, and eosinophils) may be primarily responsible for localized vasodilatation, infiltration and edema, resulting in inflammation of the PHA injection site [73,74,91]. Therefore it has been recommended that tissue biopsies from the injected areas should be recollected in order to determine specific leukocytes migration to the injected site. It has been reported that basophils are the main leukocytes responsible for inflammation after 6 h post injection [92]; while neutrophils, lymphocytes, and macrophages peak between 12 and 24 h post injection [91–94]. We also know that the number of lymphocytes present pre-treatment positively affects the number of lymphocytes infiltrating into tissue [95], suggesting that the levels of basal leukocyte populations is determines the intensity of the response when it is presented with a novel antigen. This is also supported by the findings of decreased heterophil number in peripheral blood due to rapid and intensive tissue infiltration [96]. Similarly, striped hamsters (*Cricetulus barabensis*) showed a tendency for lower neutrophil proportions in peripheral blood 24-hr after PHA stimulation [94]. For this reason, PHA inflammation can still be an overall indicator of the immune response of organisms when presented with a novel antigen, even without assessment of the specific cellular population migrating to the site.

Overall, our results were concordant with other literature reports, which have shown that variation in immune cell abundance and distribution on a seasonal basis is common [46]. The PHA index and BA were found to be negatively correlated during P1, which agrees with other literature reports [22]. The magnitude of the PHA response was not significantly different between sexes through the year in both periods examined. The PHA response only differed briefly during P1 at 6 h after injection, but the difference between sexes disappeared after 12 and 24 h. The magnitude of the PHA response was generally greater during winter, while plasma BA was higher during summer [46,47]. A possible explanation is related to the low energy requirement needed to induce the PHA response [97,98], since bats tend to forage less during the winter, achieving lower daily rates of energy intake and thus predisposing animals to a recurrent use of torpor [57]. At the same time, the high metabolic cost of prey digestion further limits animals net energy intake [99]. Furthermore, in both in-between and disregard PCA analyses, the magnitude of the PHA response was positively correlated with at least one enzymatic antioxidant activity in different basal conditions: with CAT and SOD in P1, and with GPx in P2.

Part of the immune response relies on immune cells that kill pathogens by releasing pro-oxidant compounds. Phagocytosis stimulates the so-called “respiratory burst” which results from NADPH oxidase activation, an enzyme normally inactive in resting cells. NADPH oxidase produces the superoxide free radical ($O_{2}{}^{\underline{•}}$). These reactive oxygen species can destroy microorganisms or other foreign matter [100,101]. Hence, immune cells are particularly sensitive to oxidative stress, since ROS generated outside the immune cells can affect the integral membrane function, including the cell-mediated immune reaction involving phagocyte membrane NADPH oxidase which leads to depressed immunocompetence [100]. Furthermore a continually activated immune response (i.e. chronic inflammation) may cause extensive tissue oxidative damage due to the consequent sustained ROS increase [102]. Therefore, the positive correlation between PHA response and the enzymatic activity found in our study, as well as the low carbonyl presence during winter, suggests that bats are able to maintain their redox balance while maintaining a high inflammation index.

Plasma BA during P1 was generally (D-PCA) positively related with CAT activity; however, bats with high SMI scores increased BA after the 6-hour stress. Our results agree and support the hypothesis that after short acute stress the immune response can be enhanced [55,90,103–107]. However, this occurred only during summer, when more energetic demanding processes are taking place, and no changes in BA were found during winter or autumn. This suggest that intensity of the humoral immune response to stress is highly dependent on the time of the year when it is being measured, and might explain the discrepancies found in several reported results on wild animal [54,108]. After the 12-h stress episode, BA was no different from basal levels, which suggests that after prolonged stress investment in an enhanced humoral immune response is reduced, but maintained at basal levels.

In regards to AEA defenses, our results showed that SOD and CAT in particular play an important role in *M*. *vivesi* homeostasis maintenance through the year, and that their activity was mostly related to environmental cues. The fact that protein damage had a low coefficient when testing the PCA model suggests that bats maintain redox balance and have minimum systemic damage.

Seasonal variation in the physiological variables was clearly apparent in our analyses as shown by dissimilar physiological responses presented by bats across seasons, which denoted their ability to modulate their defenses according to environmental changes and time of the year, partially supporting our hypothesis. However, winter was apparently not the most adverse stressful season as we predicted based on the low levels of AEA and carbonyl concentration measured in both periods reflecting low oxidative damage. This finding directly contrasted with reports describing high oxidative damage and high AEA during winter in birds [109]. As mentioned before, strong winds, strong surf and low T_a_ during winter may limit foraging in fish-eating Myotis promoting the use of torpor [57]. As we only sampled active bats, it is impossible to know if their low AEA activity is related to the enzymatic deployment during the respiratory burst that occurs during torpor arousal [110,111]. Either way, redox balance appeared to be undisturbed during this season, and stress did not seem to have any detrimental effect or change on their basal physiological parameters. Meanwhile, autumn was the season in which bats presented their highest SMI and AEA defenses and, as predicted, this season acted as a physiological buffer between winter and summer. Bats in summer also presented high AEA, which was strongly correlated with the elevated seasonal temperatures, to which we know *M*. *vivesi* are highly sensitive [56]. Moreover, bat reproductive state also played a major role in AEA response during this season. ROS production increases during catabolism, immune challenges, and periods of environmental stress [112]. Therefore, activities that increase metabolic demands, such as reproduction, could lead to greater ROS production, thereby imposing a higher demand for antioxidant synthesis or transport and might require the use or maintenance of repair mechanisms to avoid oxidative damage accumulation [112]. This effect was probably reflected in bat´s response to unpredicted acute stress. Bats generally responded to acute stress (both after 6 and 12 h) by enhancing their AEA activity, especially during summer and autumn, and to a lesser extent in early winter, but it was during summer when protein damage increased after stress episodes. This suggests that when energetic stress increases as a result of the simultaneous occurrence of multiple physiological challenges (i.e. humoral immune response, AEA response, thermoregulation demands due to high temperatures, and lactation) higher levels of oxidative damage may accumulate due to imbalances in oxidative stress and the capacity to mitigate such damage.

Female mammals tend to invest more energy into parental care than males, and lactation is their most energetically demanding parental endeavor [113,114]. As a consequence, it has been hypothesized that oxidative stress increases during lactation [114]. Lactating Mongolian gerbils (*Meriones unguiculatus*) had lower antioxidant defense and greater protein damage than non-reproductive females [115]. Lactating red squirrels (*Tamiasciurus hudsonicus*) had higher protein and oxidative damage, more energy demand and lower antioxidant defenses than non-reproductive females [103]. However, when lactating squirrels were supplemented with abundant food resources, they presented elevated antioxidant protection and reduced plasma protein oxidation relative to squirrels on natural (low) food resource [116]. These reports coincide with our results. During summer, most females were lactating, and even so they had increased AEA activity, concurring with a high SMI and hematocrit percentage, suggesting that high body condition and abundant resources available enable them to balance oxidative damage during this period [57,117]. However, if stress is extended for a prolonged time (e.g. 12 h), oxidative stress increases and it begins to produce substantial damage. Our study did not evaluate the degradation and replacement mechanism of oxidized proteins, so it is not possible to determine if protein damage generated after stress was accumulated or not. Even so, the antioxidant defense enhancement presumes an effective physiological response to maximize fitness. Therefore, our results suggest that *M*. *vivesi* is capable of modulating its physiological mechanism to maintain homeostasis through the year, and that individuals are highly resilient to unpredictable stress. If enough food resources are available bats would be able to oppose oxidative stress and maintain fitness even during periods of high energy demand such as lactation. These findings support the predictions of the allostasis hypothesis. Therefore, taking into account the time of the year while interpreting stress response its and integral part.
